# Factors influencing medical students in a lower-middle income country to consider psychiatry as a career option

**DOI:** 10.3389/fmed.2025.1635224

**Published:** 2025-10-22

**Authors:** Vincent I. O. Agyapong, Reham Shalaby, Gerald Agyapong-Opoku

**Affiliations:** ^1^Department of Psychiatry, Faculty of Medicine and Dentistry, University of Alberta, Edmonton, AB, Canada; ^2^Ghana Mental Health Authority, Accra, Ghana; ^3^Lilian Osborn High School, Edmonton, AB, Canada

**Keywords:** LMIC, psychiatry as a future career, psychiatry clinical training, medical students, diaspora

## Abstract

**Objective:**

To assess and identify the environmental, curriculum, teaching related factors and preconception about psychiatry that influence medical students’ attitudes toward psychiatry careers after completing psychiatry rotations.

**Methods:**

This is a cross-sectional study involving fifth- and sixth-year medical students of four public medical schools in Ghana. Data was analyzed using chi-square test and logistic regression analysis.

**Results:**

Out of 1,041 clinical year medical students from the four public medical schools in Ghana, 475 students completed survey forms and provided responses related to their preference for a psychiatry career following the completion of a clinical rotation, yielding a response rate of (45.63%). Medical students who were identified as female (OR = 1.55; 95% CI: 1.01–2.35), were in their sixth year (OR = 1.65; 95% CI: 1.06–2.58), had diaspora-based psychiatrists participate in their clinical training (OR = 1.7; 95% CI: 1.10–2.62), and had considered psychiatry careers before undertaking psychiatry clinical rotation (OR = 3.19; 95% CI: 1.73–5.87) were more likely to consider psychiatry as a future career after completing a psychiatric rotation, when compared to their respective counterparts.

**Conclusion:**

Diverse factors have affected students’ consideration of psychiatry as a future career. Health policy makers and health training institutions in low- and middle-income countries should consider designing programs that will impact positively on the preconceptions of medical students about psychiatry careers in addition reaching out to human resources abroad from within their nationals, particularly if these resources exist.

## Highlights

- Diaspora-based psychiatrists’ involvement in clinical teaching was significantly associated with medical students’ consideration of psychiatry as a career option after controlling for other factors.
- Preconception of medical students about psychiatry careers prior to undertaking a psychiatry clinical rotation was the biggest factor influencing medical students’ consideration of psychiatry as a career option.
- It is important for health policy makers in LMIC to promote stigma reduction initiatives and improving the conditions of service for psychiatrists as a way of improving the preconceptions of medical students about psychiatry careers.
- Health training institutions in LMIC should reach out to psychiatrists’ clinical teaching resources from within their nationals living abroad if these resources exist.

## Introduction

Recruiting and retention of psychiatrist workforce is a worldwide challenge affecting high-income as well as LMIC (1, 2). The World Health Organization (WHO) reported that at least half of the world’s population cannot obtain essential health services (3), and while at least 10% of the total population endure a mental health problem at any given time, only 1% of the global health workforce provides mental health care (4). According to the WHO report in 2017, the estimated rate for all the mental health workers was at 9/100,000 individuals globally, and significantly less in the low-income countries where it was below 2/100,000 individuals (5). In a particular look at the psychiatrists’ numbers, in the high-income countries there were only 11.9 psychiatrists per 100,000 people, and was far less in the low-income countries at less than 0.1 psychiatrists per 100,000 people (5).

In Ghana, the proportion of psychiatrists to the general population is 0.07 per 100,000 (6), suggesting that just about 20 psychiatrists are available for the entire population of about 28 million people which is not atypical for a LMIC. Mental health in Ghana continues to be under-resourced, with only 2.3% of the total national health budget for 2009 was allocated to mental health (7). While a modern mental health law was passed in Ghana in 2012, the Legislative Instrument (LI) needed to operationalize the law that was only passed by the Parliament of Ghana on July 2019. While the LI reiterated the need to establish a mental health levy to provide sustainable funding, the establishment itself was left to the Minister for Finance. Till date, mental health remains largely underfunded in Ghana, a situation which will be a considerable challenge for the effective implementation of an otherwise valued law (8).

In addition to the prominence of this gap in many LMICs, stigma associated with mental illness has negatively impacted the medical students’ choices for psychiatry as a career (9). The negative views about Psychiatry as a specialty were preformed even prior to formal medical training, however, they were more prominent after having clinical clerkship in Psychiatry (10). The clerks usually report perceiving dealing with patients with mental illness as dangerous, uncomfortable, stressful, overwhelming and associated with poor prognosis (9). Moreover, considering psychiatry as a career is sometimes associated with negative image from the families (11, 12) and other medical professionals (13), who perceive Psychiatry as less likely to be prestigious or financially rewarding as other specialties (10, 14).

In a research hold in the nineties, it was reported that less than 8 % of medical students were very interested in psychiatry and of those, only half percent considered it as their top choice (10). These figures seems improving over time, where in a recent survey, it was reported that 14% of medical students expressed their interest in psychiatry, nevertheless, only 4 % identified it for the first rank choice (15).

The literature suggested possible influential factors that could be attributed to the less consideration of psychiatry as a career option. These factors are probably related to the nature of training, such as duration of clerkships, problem-based learning, patient care involvement early clinical experience, or the personal characteristics of the medical trainees such as, verbal ability, general information, female gender, perception of psychiatry as more art and less science oriented (15, 16). Furthermore, clinical factors such as, lack of scientific foundation, over identifying with patient, stigma, misconception of the high violence rates expressed by psychiatric patients, poor prognosis or overwhelming, and lack of career advice, are all expressed as negative factors for considering psychiatry as a future career (17). On the other hand, the positive clinical experience, positive prognosis and patient improvement, higher level of feedback on the psychiatric practice are generally perceived as positive factors (18).

It is clear from the literature that multiple factors contribute to the interest of medical students in psychiatry both as a subject and as a career option. Not much research has however been conducted to identify the specific contribution of environmental, curriculum and teaching related factors as well as attitudes of medical student to psychiatry prior to undertaking a psychiatry rotation on their attitudes toward careers in psychiatry after completing psychiatric rotations while controlling for other factors.

In a trial to combat this gap, over the last 15 years, Ghanaian psychiatrists living and working aboard (diaspora) have been regularly involved in teaching medical students in their native country through a number of well-organized volunteer-based initiatives, in an attempt to increase the capacity of medical graduates to adequately manage psychiatric disorders. The impact of these interventions is currently being assessed as part of this study.

This study aims to assess and identify the environmental, curriculum and teaching related factors and prior interest in psychiatry before undertaking a clinical rotation in psychiatry that influence medical students’ attitudes toward psychiatry careers after completing psychiatry rotations. We hypothesize that these factors would strongly predict medical students’ positive attitudes toward careers in psychiatry.

## Methods

### Study design and setting

This study is a cross-sectional survey with quantitative methodology. The study was run in the four public medical schools available in Ghana. Each medical school runs a different schedule of psychiatry rotation during the clinical years and this range from 4 to 6 weeks during the 4th or 5th year to 2–3 weeks each during the 5th and 6th years. In addition, each school has its independent curriculum and there are varying degrees of involvement of diaspora-based psychiatrists in clinical teaching in the schools.

### Study participants and data collection

Based on the lack of existing literature and validated assessment tools specific to the study objectives, an English self-administered questionnaire (Supplementary Appendix 1) with Likert scale responses was designed by the researchers to assess the key objective of the study. The Likert responses offered scores of 1–4 for each question, where one represented woefully inadequate, two represented inadequate, three represented somewhat adequate and four represented adequate. The four responses were collapsed during analysis into two main responses: *Adequate/somewhat adequate* and *Inadequate/woefully inadequate*. The 22-item questionnaire assessed variables included gender and clinical year. It also included the medical students perceptions about psychiatrist to patient ratio, nurse to patient ratio, infrastructure for mental health, infrastructure for clinical training of the medical students, psychiatrists to medical students’ ratio for clinical training of the medical students, depth of psychiatry curriculum, depth of clinical training during psychiatric rotations, contact time with psychiatrists during rotations, length of psychiatric rotation undertaken, depth of experience gained and Ghanaian diaspora based psychiatrists participated in clinical training. It also included a question on their attitude toward Psychiatry prior to undertaking clinical rotation.

The questionnaire was pretested on five randomly selected potential study participants. They were revised based on the results of the pre-test to give it a better structure and to introduce more Likert scales for some responses. They generally took 10 min to complete, and no monetary or other incentives were provided to the respondents.

All surveys were conducted in paper and pen format and were administered by an executive officer from the Federation of Ghana Medical Students Association (FGMSA) in each of the four medical schools who worked as research associates and collaborated with the lead author on data collection. Information leaflets were initially distributed to all 1,041 fifth- and sixth-year medical students at the various student hostels on the university campuses. The informed leaflet included a statement to the effect that consent would be implied if the student completed and returned the survey questionnaire to the FGMSA officer.

### Data analysis

Data were analyzed using descriptive statistics, chi square and logistic regression analysis using SPSS version 20. Data were derived from categorical responses from a survey questionnaire. Descriptive data for the different categorical variables reported by the medical students were compared against their attitudes toward psychiatry as a future career after completing a psychiatric rotation using chi-square test. Variables with a statistically significant association with medical consideration of psychiatry as a career option on univariate analysis, were included in a logistic regression analysis. Before performing logistic regression, correlational analysis was performed to determine any strong inter-correlations (Spearman’s correlation coefficient of 0.7–1.0 or −0.7 to −1.0) among predictor variables. Odds ratios and confidence intervals from the binary logistic regression analysis were examined to determine the association between each variable in the model and medical students’ consideration of psychiatry as a career option after their psychiatry rotation, controlling for the other variables. We presented results in numbers and percentages and a two-tailed significance value of 0.05 or less was deemed as the acceptable statistically significance level.

The raw data supporting the conclusions of this manuscript are stored on a password protected computer in possession of the corresponding author and will be made available without reservations to any qualified researcher, subject to appropriate ethics or legal requirements.

## Results

In all, 475 medical students out of 1,041 clinical year students from the four public medical schools in Ghana returned completed survey forms, giving a response rate of 45.63% for this study. Participants included 250 males (52.63%) and 225 females (47.37%). Table 1 shows the association between gender, clinical year of the respondents, respondents’ perceptions about the various environmental, curriculum and teaching related factors as well as their preconception toward careers in psychiatry and their attitudes toward careers in psychiatry after completing their psychiatry clinical rotation.

**Table 1:** Chi-square test of association between attitude toward psychiatry after training and different sociodemographic, environmental, curriculum, and training related variables and preconception.

Variable		Attitude toward psychiatry after rotation		Total	χ^2^	*p*-value
		Considered a career in psychiatry	Was not decided/had ruled out career in Psychiatry			
Gender	Female	86 (38.2%)	139 (61.8%)	225	3.94	**0.047**
	Male	74 (29.6%)	176 (70.4%)	250		
Clinical year	Sixth year	106 (38.1%)	172 (61.9%)	278	5.93	**0.015**
	Fifth year	54 (27.4%)	143 (72.6%)	197		
Psychiatrist to patient ratio	Adequate or somewhat adequate	17 (26.6%)	47 (73.4%)	64	1.95	0.162
	Inadequate or woefully inadequate	143 (35.5%)	260 (64.5%)	403		
Nurse to patient ratio	Adequate or somewhat adequate	31 (38.8%)	49 (61.2%)	80	0.834	0.361
	Inadequate or woefully inadequate	128 (33.4%)	255 (66.6%)	383		
Infrastructure for mental health	Adequate or somewhat adequate	16 (53.3%)	14 (46.7%)	30	5.13	**0.023**
	Inadequate or woefully inadequate	144 (33%)	292 (67%)	436		
Infrastructure for clinical training of the medical students	Adequate or somewhat adequate	60 (36.4%)	105 (63.6%)	165	0.610	0.435
	Inadequate or woefully inadequate	99 (32.8%)	203 (67.2%)	302		
Psychiatrists to medical students’ ratio for clinical training of the medical students	Adequate or somewhat adequate	40 (32.3%)	84 (67.7%)	124	0.154	0.694
	Inadequate or woefully inadequate	118 (34.2%)	227 (65.8%)	345		
Depth of psychiatry curriculum	Adequate or somewhat adequate	129 (37.7%)	213 (62.3%)	342	8.49	**0.004**
	Inadequate or woefully inadequate	30 (23.4%)	98 (76.6%)	128		
Depth of clinical training during psychiatric rotations	Adequate or somewhat adequate	123 (38.7%)	195 (61.3%)	318	10.48	**0.001**
	Inadequate or woefully inadequate	37 (23.7%)	119 (76.3%)	156		
Contact time with psychiatrists during rotations	Adequate or somewhat adequate	105 (38.3%)	169 (61.7%)	274	4.1	**0.044**
	Inadequate or woefully inadequate	54 (29.2%)	131 (70.8%)	185		
Length of psychiatric rotation undertaken	Adequate or somewhat adequate	111 (39.8%)	168 (60.2%)	279	10.79	**0.001**
	Inadequate or woefully inadequate	49 (25.3%)	145 (74.7%)	194		
Depth of experience gained	Adequate or somewhat adequate	116 (37.4%)	194 (62.6%)	310	6.31	**0.012**
	Inadequate or woefully inadequate	42 (25.9%)	120 (74.1%)	162		
Ghanaian diaspora-based psychiatrists participated in clinical training	Yes	94 (42)	130 (58)	224	12.93	**0.000**
	No	64 (26.2)	180 (73.8)	244		
Attitude toward psychiatry prior to undertaking clinical rotation	Had considered a career in psychiatry	35 (57.4%)	26 (42.6%)	61	17.59	**0.000**
	Was not decided or had ruled out a career in psychiatry	125 (30.2%)	289 (69.8%)	414		

Bold values indicate significance.

Table 1 shows that as many as 10 factors showed significant association with attitudes toward psychiatry after the clinical rotation. For example, female medical students and sixth year clinical students were significantly more likely to consider psychiatry careers after completing a psychiatry rotation compared to their male counterparts and fifth year medical students, respectively.

The only environmental factor which showed a significant association with the attitudes of the medical students toward careers in psychiatry was “infrastructure for mental health care,” where medical students who perceived the infrastructure as at least somewhat adequate were more likely to consider psychiatry careers compared to those who thought the infrastructure was inadequate.

Table 1 also shows that all the curriculum and teaching related factors as well as preconception of the medical students showed a significant association with the attitudes of the medical students toward careers in psychiatry after completing their psychiatry rotations. Medical students who ranked the depth of psychiatry curriculum, depth of clinical training during psychiatric rotations, contact time with psychiatrists during rotations, length of psychiatric rotation undertaken and the depth of experience gained at least as somewhat adequate were more likely to consider careers in psychiatry compared to those who ranked these variables as inadequate.

Similarly, those who reported that Ghanaian diaspora-based psychiatrists participated in clinical training for their psychiatry rotation and those who had considered careers in psychiatry prior to undertaking rotations in psychiatry were more likely to consider psychiatry as a career option after their psychiatry rotation. This is compared to those who reported that diaspora-based Ghanaians did not participate in their clinical teaching and those who had ruled out psychiatry as a career option before undertaking rotations in psychiatry, respectively.

Spearman correlation analysis was run yielding no strong correlation present among the variables which showed significant associations with the attitudes of the medical students toward careers in psychiatry after their psychiatry rotations, so all 10 variables were entered into a logistic regression model as shown in Table 2.

**Table 2:** Logistic regression of predictor variables and their association with the attitudes of medical students toward psychiatry careers after completing a clinical rotation.

Independent variable	*B*	Standard error	*p-*value	Odd’s ratio	95% CI	
					Lower	Upper
Gender (female)	0.435	0.215	**0.04**	1.545	1.01	2.35
Clinical year (6th)	0.502	0.228	**0.03**	1.65	1.06	2.58
Infrastructure for mental health (adequate or somewhat adequate)	0.743	0.455	0.10	2.102	0.862	5.124
Depth of psychiatry curriculum (adequate or somewhat adequate)	0.331	0.309	0.28	1.392	0.761	2.549
Depth of clinical training during psychiatric rotations (adequate or somewhat adequate)	0.355	0.282	0.21	1.426	0.820	2.479
Length of psychiatric rotation undertaken (adequate or somewhat adequate)	0.517	0.295	0.08	1.677	0.941	2.990
Contact time with psychiatrists during rotations (adequate or somewhat adequate)	−0.046	0.258	0.86	0.955	0.576	1.584
Depth of experience gained (adequate or somewhat adequate)	−0.069	0.294	0.81	0.933	0.524	1.662
Ghanaian diaspora-based psychiatrists participated in clinical training (yes)	0.527	0.222	**0.02**	1.694	1.097	2.617
Attitude toward psychiatry prior to undertaking clinical rotation (had considered a career in psychiatry)	1.159	0.311	**0.00**	3.188	1.732	5.868
Constant	−1.392	0.301	0.00	0.249		

Bold values indicate significance.

The full model containing all 10 predictor variables was statistically significant with [X^2^(10) = 54.51, *p* < 0.00]. The model as a whole explained between 11.7 and 16.1% (Cox and Snell R2, Nagelkerke R2, respectively) of the variance in student’s consideration of psychiatry as a future career following the completion of psychiatry rotations and correctly classified 70.3% of cases.

As shown in Table 2, only four of the 10 predictor variables made unique statistically significant contributions to the model. With an odds ratio of 1.54, female medical students were one and half times more likely to consider psychiatry as a future career after completing a psychiatric rotation, controlling for other variables in the model. Similarly, sixth year medical students were one and half times more likely to consider psychiatry as a future career after completing a psychiatric rotation than fifth year medical students, controlling for other variables in the model.

Furthermore, medical students who had diaspora-based psychiatrists participate in their clinical training were more than one and a half times more likely to consider careers in psychiatry after their clinic training compared to those who did not receive diaspora-based training, controlling for other factors in the model. The biggest contributor to the logistic regression model was *the prior attitudes of the students toward psychiatry* careers. After controlling for other factors, medical students who had considered psychiatry careers before undertaking a psychiatry clinical rotation were about three times more likely to consider careers in psychiatry after their psychiatry rotation compared to those who had ruled out careers in psychiatry before their psychiatry rotation.

## Discussion

With significantly low numbers of medical students globally considering psychiatry as a future career relative to other medical specialties, this cross-sectional study aimed to assess the different potential factors related to medical students’ considerations of psychiatry as a future career upon completing their psychiatry rotations, using logistic regression analysis. Similar approach was performed in related studies (19, 20), where a significant attention was given to the interest of medical students in psychiatry after receiving the clinical training, either through the regular clinical placement or an elective training (20–22). The regression model in our study explained 16.1% of the variance, compared to 11% in another study (23), suggesting the inclusion of more predictive values in our model.

Our study suggests that, after controlling for other factors, there was a significant association between female gender and considering psychiatry as a future career. This was consistent with most of other studies (19, 21, 23–25), however one study reported no difference in preference for a psychiatry career based on gender of the medical students (26). Additionally, this was further argued in the report of a large national survey hold in the UK, where the year of graduation was suggested to middle this relationship between gender and choosing Psychiatry as a career, for example in the third year, this difference was significantly evident, compared to year one after graduation, where the difference was nullified (27). Research indicates that female medical students are less likely to anticipate earning a high income and are more inclined to prioritize work-life balance, factors that may contribute to a stronger interest in psychiatry (28). Gender has also been shown to shape career decision-making, with female students’ expectations and experiences in mentoring relationships playing an important role (29). This suggests that increasing the availability of female mentors in psychiatry could encourage more female medical students to pursue careers in the field.

Clinical year of study was a significant determinant for students’ interests in psychiatry after completing their clinical rotation. Sixth year medical students expressed more interest in a psychiatry career. This was not typically consistent with what was reported in other studies, which showed no difference among different years of clinical training at medical school and a preference for psychiatry (20, 26) with one of the studies suggesting that older students had a higher tendency to not opt for psychiatry as a future career (20). Other related factors that could affect Psychiatry as a career choice, one review reported that one and 3 years after qualification, 4–5% of doctors in the UK opt for psychiatry (27).

Our study reported that the strongest predictor for a consideration of a psychiatry career after completing a psychiatry rotation was medical students’ preconception about a psychiatry career prior to undertaking the psychiatry rotation. This is consistent with results of a national survey which concluded that clear and early choice for psychiatry while a student is a significant predictor of a subsequent career in the specialty (27). This tendency could have been formulated even before medical school admission and perceived as impactful for considering the future career in psychiatry (20). Similarly, our results suggested that, while education environment is effective in changing students’ perception of psychiatry as a discipline, it did not appear to be an effective determinant for choosing psychiatry as a future career; this was consistent with what was reported in the literature (22).

In a survey of factors affecting students’ preference for a career in psychiatry, length of psychiatry rotation was perceived as an important predictor for increasing their interest in psychiatry as a future career, especially if it is provided for more than a month (26). Another survey also reported as association between the number of weeks for psychiatry training and medical students’ consideration of psychiatry as a career option (20). Consistent with these studies, our study has established the positive perceptions of length of psychiatric rotation undertaken as a positive predictor of medical students’ consideration of careers in psychiatry, although this association was not statistically significant when other factors are controlled for in the logistic regression model.

Clinical teaching during psychiatric rotation, depth of experience gained, and positive placement experience could be sometimes satisfying for medical students regardless of their particular interest in psychiatry (18). This is again consistent with observations in our study which highlighted that students’ positive perception of the experience gained and the depth of both curriculum and clinical training after having psychiatry clinical rotation were positively associated with a propensity to consider psychiatry as a future career, although again, these associations were nullified when considering other factors.

### Initiatives undertaken to overcome the global problem of deficiency of psychiatrists

Where only four to 7 % of medical students typically opt for a career in psychiatry (30), there are more than 10 % for example prefer other specialties like General Surgery or Ophthalmology (31). This discrepancy resulted in a wider treatment gap for mental health patients. For example, the treatment gap for schizophrenia and other psychoses is over 90% (32) and typically, 75% of individuals diagnosed with non-psychotic mental illnesses receive no care at all (33). It is also suggested that in the underserviced communities that lack enough medical facilities, many patients opt to seek help from informal health services such as traditional and faith-based practitioners (34).

For this, several initiatives have been implemented in the past to stimulate the medical students’ interest in Psychiatry as a subject as well as a future career. In one study, it was suggested that governmental incentive support systems offered for doctors who opt to train in psychiatry could be an effective strategy toward stimulating the interest of medical students in psychiatry (35). The literature on undergraduate psychiatric clerkships also suggests that early exposure to the clinical experience, which is relatively uncommon in psychiatry and longer clerkships correlate with more positive attitude by students to patients with psychiatric illnesses (9, 16, 36). However longer clerkships did not show significant correlation with an improvement in the interest of medical students in psychiatry as a career option (9, 36). Interestingly, it was found that an outpatient clerkship may have a more positive effect on career choice than rotation through other settings (37).

In Ghana, an evaluation of an annual public speaking competition to promote psychiatry as a career option for medical students, revealed that a significantly high proportion of those who participated in at least one competition-related activity perceived the competition as a stimulus for their interest in psychiatry, compared to those who did not participate (2).

While it is described as “ambiguous” whether to focus on the educational aspect of psychiatry as a subject or clinical rotation before graduation or instead increasing the postgraduate incentives, in terms of work placements or better salaries (26, 38), innovative teaching strategies such as diaspora based psychiatrists sharing in clinical training of medical students and enhancing the psychiatry curriculum even at the preclinical years of medical school (39) have been suggested as potentially effective in enhancing the global experience and perceptions of medical students about psychiatry as a subject and as a career option. Style of teaching and traits of professors of psychiatry were also considered important factors for choosing psychiatry as a future specialty (38, 40). Senior versus junior clinicians teaching during clinical placements also could have a differential impact upon the choice of the specialty (20). Similarly, having the usual ward clinical training were not seen as promoters for students’ interest in career in psychiatry, and might have a negative impact on medical students that could skew their preference for psychiatry in the future (20, 21, 26, 41). Interestingly, factors related to the training curriculum—such as its length and depth—were not found to be statistically associated with the decision to consider psychiatry as a future career. This finding may be attributable to the relatively small sample size, as well as the limitations of the questionnaire, which lacked a qualitative component that could have explored this relationship with greater depth and nuance.

Finally, the literature suggests that tapping into the vast potential and opportunities offered by diaspora-based nationals to address unmet human resource needs for clinical teaching in LMIC could promote medical students’ aspiration and interest in psychiatry (21, 38). Diaspora based clinicians possess many valuable potentials including ability to transfer evidence-based practice, bridging cultural differences and training of native healthcare workers within common-language communication settings, thus linking lower medium to high income countries (42, 43). As well, they can use their “in-country networks” to create effective “international partnerships” helping to promote their native countries’ healthcare system (42) and might provide a substantial success in building up research capacity in their home countries (44).

Our study has a number of important limitations. First, the survey tool used was not a validated instrument and therefore may lack external validity. Second, we achieved a response rate of only 45.62%, which raises the possibility of self-selection bias. It is plausible that students with a pre-existing interest in psychiatry, or those with stronger views about their rotation experiences, were more likely to respond, potentially skewing the results toward a more favorable or unfavorable impression of psychiatry than might be present across the entire student body. Third, although our findings provide useful insights into the attitudes of Ghanaian medical students, the results may not be generalizable to all medical students in Ghana or to other LMIC contexts, given differences in training environments, exposure to psychiatry, and availability of diaspora engagement. Finally, while our study suggested that involving diaspora-based psychiatrists in teaching during clinical rotations could enhance the likelihood of students considering careers in psychiatry, we did not explore the underlying reasons for this association. To address this gap, further studies incorporating qualitative methodologies are planned by the authors to explore these dynamics in greater depth.

Notwithstanding these limitations, our study present important and novel findings in the context of LMIC which can assist health policy planners and medical training institutions to formulate policies and programs that will increase the number of psychiatry residents and thereby increase the psychiatrist-to-patient ratio in Ghana and other LMIC.

Static factors that were significantly associated with medical students’ consideration of psychiatry as a career option included female gender, senior year in medical school and a favorable preconceived choice or consideration for a psychiatry career prior to undertaking a psychiatry clinical rotation. The only modifiable factor associated with a favorable consideration of psychiatry as a career option after completing a clinical rotation was the involvement of diaspora-based psychiatrists in clinical teaching. In addition to their direct contributions to medical student teaching, diaspora-based psychiatrists can play a significant role in policy advocacy by leveraging their expertise, international networks, and experiences across health systems to inform mental health policy in their home countries. They are uniquely positioned to advocate for the prioritization of psychiatry within national health agendas and can help align local training programs with global standards while remaining sensitive to cultural contexts. Furthermore, diaspora psychiatrists can contribute to curriculum development by introducing innovative, evidence-based teaching methods and integrating emerging topics in psychiatry, such as global mental health, transcultural psychiatry, and community-based interventions. Their dual familiarity with both high-resource and low-resource health systems enables them to contextualize curricula in ways that are both aspirational and feasible for LMIC settings. Specifically, governments and health policy makers can address human resource gaps utilizing expertise from its nationals abroad in the following ways:

- *Leverage diaspora expertise for training*—Actively engage diaspora-based psychiatrists in clinical teaching, mentoring, and supervision to supplement local workforce shortages and provide diverse perspectives in psychiatric training.
- *Integrate diaspora input into policy advocacy*—Involve diaspora psychiatrists in shaping national mental health agendas by drawing on their international networks and cross-system experiences to advocate for psychiatry’s prioritization in health policy.
- *Support curriculum innovation*—Collaborate with diaspora psychiatrists to revise and enrich psychiatry curricula, incorporating global mental health, transcultural psychiatry, and community-based approaches that are both evidence-based and contextually relevant.
- *Strengthen research capacity*—Utilize diaspora partnerships to build sustainable research collaborations that enhance local capacity for psychiatric research and innovation.
- *Address stigma and misconceptions*—Launch awareness campaigns, endorsed by health policymakers and academic leaders, to challenge negative perceptions of psychiatry among families, peers, and medical professionals, emphasizing its scientific foundation, societal relevance, and career viability.
- *Create structured diaspora engagement mechanisms*—Establish formal platforms (e.g., visiting professorships, short-term teaching fellowships, virtual teaching programs) that make it easier for LMIC institutions to regularly access diaspora expertise.

Taken together, these broader opportunities for contributing suggest that diaspora engagement has the potential not only to enhance the quality of psychiatry training but also to strengthen national mental health policies and modernize medical education, thereby fostering long-term system-level improvements. It is therefore important for health policy makers and health training institutions in LMIC that have limited numbers of psychiatrists to reach out to psychiatrist clinical teaching resources from within their nationals living abroad if these resources exist. Health policy makers can also improve the negative image from the families (11, 12) as well as other medical professionals (13) about psychiatry. This will also dispel perceptions that psychiatry is less prestigious or financially rewarding as compared to other specialties (10, 14) and hopefully increase the number of medical students considering psychiatry as a career option even before their clinical rotation.

## Data Availability

The raw data supporting the conclusions of this article will be made available by the authors, without undue reservation.
