# Supplementary material for: Factors influencing medical students in a lower-middle income country to consider psychiatry as a career option
Source: Front Med (Lausanne). 2025 Oct 22;12:1635224. doi: 10.3389/fmed.2025.1635224 (PMC12586103; doi:10.3389/fmed.2025.1635224)
Supplement: Supplementary file 1 [file Table_1.docx]

**Appendix 1: Factors influencing medical students in a lower-middle income country to consider psychiatry as a career option- survey questions**

***Survey Questions for Medical Students***

1. **Which medical school do you attend?**

KNUST SMS_ UGMS_ UDS SMS_ UCC SMS_

1. **Which clinical year are you in currently?**

5^th^ Year_ 6^th^ Year_

1. **Gender:** Male _ Female_
2. **How do you perceive the psychiatrists to patient ration in Ghana?**

Adequate_ Somewhat adequate_ Inadequate_ Woefully inadequate_

1. **How do you perceive the nurse/community mental health worker to patient ration in Ghana?**

Adequate_ Somewhat adequate_ Inadequate_ Woefully inadequate_

1. **How do you perceive the infrastructure for mental health in Ghana?**

Adequate_ Somewhat adequate_ Inadequate_ Woefully inadequate_

**Undergraduate Psychiatry Rotation**

1. **Have you yet done a clinical rotation in Psychiatry:** Yes_ No_
2. **How do you perceive the infrastructure for the training of medical students in your school?**

Adequate_ Somewhat adequate_ Inadequate_ Woefully inadequate_

1. **How do you perceive the psychiatrists to medical student/resident ratio for the training of medical students in your school?**

Adequate_ Somewhat adequate_ Inadequate_ Woefully inadequate_

1. **How do you perceive the length of the psychiatric rotation for medical students in your school?**

Adequate_ Somewhat adequate_ Inadequate_ Woefully inadequate_

1. **How do you perceive the depth of the psychiatry curriculum for clinical year students in your school?**

Adequate_ Somewhat adequate_ Inadequate_ Woefully inadequate

1. **How do you perceive the depth of clinical teaching during the psychiatric rotations for medical students in your school?**

Adequate_ Somewhat adequate_ Inadequate_ Woefully inadequate

1. **How do you perceive the contact time with psychiatrists during the psychiatric rotations for medical students in your school?**

Adequate_ Somewhat adequate_ Inadequate_ Woefully inadequate

1. **Did you perceive the length of the psychiatric rotation you undertook as enough to give you adequate exposure to psychiatry?**

Adequate_ Somewhat adequate_ Inadequate_ Woefully inadequate

1. **How do you perceive the depth of the experience you gained during the psychiatric rotation for medical students in your school?**

Adequate_ Somewhat adequate_ Inadequate_ Woefully inadequate_

1. **Did Ghanaian psychiatrists who are based in diaspora participate in the clinical teaching during your psychiatry clinical rotation?** Yes_ No_
2. **Does or will the participation of Ghanaian diaspora based psychiatrists in the teaching of the psychiatric curriculum for medical students stimulate your interest in psychiatry as a subject?**

Most certainly_ Certainly_ Not sure_ Not really_ Not at all_

1. **Does or will the participation of Ghanaian diaspora based psychiatrists in the teaching of the psychiatric curriculum for medical students stimulate your interest in psychiatry as career choice?**

Most certainly_ Certainly_ Not sure_ Not really_ Not at all_

1. **Should the participation of Ghanaian diaspora based psychiatrists in the teaching of the psychiatric curriculum for medical students in Ghana be actively encouraged?**

Most certainly_ Certainly_ Not sure_ Not really_ Not at all_

1. **What was your attitude towards a career in psychiatry before you undertook your psychiatric rotation?**

**C**onsidered a career in Psychiatry_

Ruled out a career in Psychiatry_

Was not decided on a career in Psychiatry_

1. **What was your attitude towards a career in psychiatry after you undertook your psychiatric rotation?**

Considered a career in Psychiatry_

Ruled out a career in Psychiatry_

Still not decided on a career in Psychiatry_

1. **Please suggest ways to improve on the structure, content or mode of delivery of the undergraduate psychiatry curriculum in other for it to better stimulate the interest of Ghanaian medical students in psychiatry**.

____________________________________________________________________________________________________________________________________________________________________________________________________________________________________________________________________________________________________________________
